# Cost-Effective and Selective Fluorescent Chemosensor (Pyr-NH@SiO_2_ NPs) for Mercury Detection in Seawater

**DOI:** 10.3390/nano12081249

**Published:** 2022-04-07

**Authors:** Shahid Ali, Muhammad Mansha, Nadeem Baig, Safyan Akram Khan

**Affiliations:** 1 Center of Research Excellence in Nanotechnology, King Fahd University of Petroleum and Minerals, Dhahran 31261, Saudi Arabia; s.ali@kfupm.edu.sa; 2 Interdisciplinary Research Center for Hydrogen and Energy Storage, King Fahd University of Petroleum and Minerals, Dhahran 31261, Saudi Arabia; manshachohan@kfupm.edu.sa; 3 Interdisciplinary Research Centre for Membranes and Water Security, King Fahd University of Petroleum and Minerals, Dhahran 31261, Saudi Arabia; nadeembaig@kfupm.edu.sa

**Keywords:** pyrene, peptide coupling, silica NPs, fluorescent nanomaterials, mercury sensing, seawater

## Abstract

The release of mercury into the environment has adverse effects on humans and aquatic species, even at very low concentrations. Pyrene and its derivatives have interesting fluorescence properties that can be utilized for mercury (Hg^2+^) ion sensing. Herein, we reported the highly selective pyrene-functionalized silica nanoparticles (Pyr-NH@SiO_2_ NPs) for chemosensing mercury (Hg^2+^) ions in a seawater sample. The Pyr-NH@SiO_2_ NPs were synthesized via a two-step protocol. First, a modified Stöber method was adopted to generate amino-functionalized silica nanoparticles (NH_2_@SiO_2_ NPs). Second, 1-pyrenecarboxylic acid was coupled to NH_2_@SiO_2_ NPs using a peptide coupling reaction. As-synthesized NH_2_@SiO_2_ NPs and Pyr-NH@SiO_2_ NPs were thoroughly investigated by ^1^H-NMR, FTIR, XRD, FESEM, EDS, TGA, and BET surface area analysis. The fluorescent properties were examined in deionized water under UV-light illumination. Finally, the developed Pyr-NH@SiO_2_ NPs were tested as a chemosensor for Hg^2+^ ions detection in a broad concentration range (0–50 ppm) via photoluminescence (PL) spectroscopy. The chemosensor can selectively detect Hg^2+^ ions in the presence of ubiquitous ions (Na^+^, K^+^, Ca^2+^, Mg^2+^, Ba^2+^, Ag^+^, and seawater samples). The quenching of fluorescence properties with Hg^2+^ ions (LOD: 10 ppb) indicates that Pyr-NH@SiO_2_ NPs can be effectively utilized as a promising chemosensor for mercury ion detection in seawater environments.

## 1. Introduction

Mercury poses extreme risks to human health including brain and neurological damage, birth deformities, kidney damage, digestive system problems, memory loss, and language impairments [1,2]. As an established environmental pollutant, mercury is found in its elemental (Hg^0^) and ionic (Hg^+^, Hg^2+^) forms in the atmosphere and water, respectively. Unfortunately, mercury emission in the environment has tremendously increased since the beginning of industrial era [3,4], where the average mercury levels in the atmosphere are enhanced three to six folds higher than pre-industrial estimates. Manifolds of the annual surge of mercury discharge in the environment pose serious threats. Therefore, there is a great desire to design reliable methods for its sensitive and selective detection in real environments, especially seawater.

Various analytical techniques can be engaged to monitor Hg^2+^ concentration, such as atomic absorption spectroscopy (AAS), inductively coupled plasma-mass spectrometry (ICP-MS), plasma-atomic emission spectrometry (AES), gas chromatography (GC), and reversed-phase high-performance liquid chromatography (HPLC). However, these techniques require expensive, specialized, cumbersome sample preparations, and bulky laboratory equipment that make it difficult to adapt for remote sensing applications. Therefore, there is a clear urgency to develop cost-effective, sensitive, selective, and more importantly, convenient, portable sensors that can detect the existence of Hg^2+^ ions up to parts per billion (ppb) levels of mercury. Recently, several reports have been published on the development of solid-state sensors for Hg^2+^ ions detection in an aqueous phase [5,6,7]. Among various techniques that allow low-level detection of mercury, optical methods based on fluorescence sensing have gained much attention [8,9,10,11]. The comparison indicates that fluorescence-based chemical sensors offer rapid analysis, better sensitivity, low limit of detection, and high selectivity for investigation of environmental pollutants even if it existed at very low concentrations [12,13,14]. This is the prime reason that researchers preferred to develop fluorescence-based sensors.

Researchers have demonstrated fluorescence-based Hg^2+^ sensors using nanoparticles, nanoclusters, quantum dots, and nanorods [15,16,17,18]. Gold nanoparticles (Au-NPs) possess strong molar absorptivity and are utilized as super-quenchers [19]. Therefore, Cho et al. [20] developed a mercury sensor made up of porphyrin-functionalized Au–SiO_2_ NPs, which showed strong fluorescent properties (red-colored). Upon the addition of Hg^2+^ ions, a turn-off/quenching phenomenon occurred, giving the green fluorescence color (LOD: 1.2 ppb). Similarly, metal nanoclusters, nanoparticles, and quantum dots have adjustable narrow emission spectra, long luminescence lifetimes, high quantum yields, and resistance from photo-bleach [21,22] that make them better optical sensors. However, the gold nanoclusters (Au-NCs) have great potential to exhibit photoluminescence and there is a direct relationship between the wavelength of emission and the size of nanoclusters [23].

Among the broad varieties of available fluorophores, pyrene and its derivatives have interesting fluorescence properties, such as long lifetime, high quantum yield, large Stokes shift, expanded π electron delocalization, and excellent photostability [24]. However, the pyrene derivatives and many other organic compounds absorb light in the UV region, which may complicate the analysis and limit their use for optical detection. The fluorescence quenching of pyrene or its derivative fluorophores mostly occurred in the presence of various quenchers, e.g., heavy metals, anionic species, and NPs, where the energy is transferred either through the bond or from space energy transfer between the pyrene and the quenchers [25]. In this regard, Manandhar et al. [26] demonstrated a molecular butterfly from a pyrene-based triazole ligand, which self-assembles in the presence of ZnCl_2_. The free ligand monomer showed the emission bands at 382 and 402 nm with a broad shoulder peak at 420 nm. However, the complex exhibits strong excimer emission at 410 nm upon the addition of ZnCl_2_ solution. Similarly, Wang et al. [27] developed a pyrene-thymine dyad that depicts a strong pyrene excimer selective emission in the presence of Hg^2+^ ions without interfering with any other metal ions. This pyrene-thymine dyad acted as a “turn-on” fluorescent sensor for Hg^2+^ ions. Moreover, Zhang et al. prepared Py-SiO_2_ core-shell NPs by making a receptor of hydrazone moiety for Hg^2+^ ions using 4-hydroxy-benzaldehyde and 1-pyrenecarboxaldehyde followed by the addition of 3-(triethoxysilyl)-propyl isocyanate as a pseudo electrophile. The silane part was further reacted with tetraethyl orthosilicate to produce silica decorated with pyrene stuff [28]. Several reports have been published to develop fluorescence sensors for mercury detection at trace levels in aquatic environments. However, the development of portable, cost-effective, highly sensitive/selective, easy to synthesize, and fabricate fluorescence sensors is ongoing for mercury analysis in real environments.

In this work, pyrene-attached silica nanoparticles (Pyr-NH@SiO_2_ NPs) were synthesized in two steps instead of a multi-step synthesis protocol. First, amino-functionalized silica nanoparticles (NH_2_@SiO_2_ NPs) were prepared by the hydrolysis of tetraethyl orthosilicate (TEOS) followed by the condensation with 3-aminopropyltriethoxysilane (APTES), which in turn covalently attached with 1-pyrenecarboxylic acid. The synthesis chemistry and detailed characterization of NH_2_@SiO_2_ NPs and Pyr-NH@SiO_2_ NPs have been described. The Pyr-NH@SiO_2_ NPs were investigated as a fluorescent chemosensor for Hg^2+^ ion detection in a real seawater sample. Finally, the sensitivity and selectivity of the developed sensor were demonstrated.

## 2. Materials and Methods

### 2.1. Materials

All the chemicals and reagents, including cetyltrimethylammonium bromide (CTAB, ≥98%, Sigma-Aldrich, St. Louis, MO, USA), sodium hydroxide (≥98%, Fluka, Hampton, NH, USA), tetraethyl orthosilicate (TEOS, ≥99%, Sigma-Aldrich), 3-aminopropyl)triethoxysilane (APTES, 99%, Sigma-Aldrich), 1-pyrenecarboxylic acid (97%, Sigma-Aldrich), hydroxy benzotriazole (≥97%, Sigma-Aldrich), 1-ethyl-3-(3-dimethyl aminopropyl)carbodiimide hydrochloride (≥99%, Sigma-Aldrich), triethylamine (>99%, Sigma-Aldrich), ethyl acetate (≥99.7%, Honeywell, Charlotte, NC, USA), were purchased and utilized without further purification.

### 2.2. Synthesis of Silica Nanoparticles (NH_2_@SiO_2_ NPs)

The synthesis of silica NPs was carried out by the modified Stöber’s method using silane precursors [29,30]. In a typical synthesis, CTAB surfactant (2.0 g, 5.48 mmol) was dissolved in deionized water (25 mL) under vigorous stirring and transferred into a round-bottom flask containing a solution of NaOH (1.40 g, 35 mmol) and deionized water (100 mL). Then, 25 mL of ethyl acetate was added to the reaction mixture and continued stirring for 10 min followed by the addition of TEOS (3.20 mL, 14.43 mmol). The reaction contents were stirred for another 40 min at room temperature, followed by the addition of APTES (2.10 mL, 8.97 mmol), and kept the reaction contents to stir overnight. The mixture was centrifuged at 10,000 rpm to separate the amino-functionalized silica nanoparticles. The product was washed thrice with deionized water and twice with absolute ethanol to remove the surfactant and impurities, respectively. As-synthesized silica NPs having a small amount of ethanol were transferred into a petri dish and allowed to evaporate the solvent in a vacuum oven at 60 °C. The fine white powder of silica (**NH_2_@SiO_2_ NPs**) was formed having a very good experimental yield (~80%). FTIR (neat): ν(cm^−1^) = 3444, 2922, 2852, 1643, 1553, 1471, 1056, 785, 451. ^1^H-NMR (400 MHz, DMSO): δ = 1.243 (m, 2H), 2.33 (t, 2H), 2.67 (t, 2H).

### 2.3. Synthesis of Pyrene-Attached Silica Nanoparticles (Pyr-NH@SiO_2_ NPs)

As-synthesized amino-functionalized silica NPs (0.200 g) and 1-pyrenecarboxylic acid (0.300 g, 1.21 mmol) were taken into a dried round-bottom flask (50 mL) followed by the addition of hydroxybenzotriazole (0.210 g, 1.55 mmol) and 1-ethyl-3-(3-dimethyl aminopropyl)carbodiimide hydrochloride (0.232 g, 1.21 mmol). Subsequently, anhydrous chloroform (20 mL) was added into the flask and stirred the reaction mixture. Then, triethylamine (0.356 mL, 2.61 mmol) was added into the reaction mixture and continued stirring at room temperature for 24 h. After completion of the reaction, the flask contents were transferred into a separatory funnel and ethyl acetate (40 mL) was added. The product was washed twice with NaOH solution (1M, 20 mL) and deionized water to remove the unwanted coupling reagents as well as unreacted 1-pyrenecarboxylic acid. Finally, the organic solvent was removed using a rotary evaporator to get the final product (**Pyr-NH@SiO_2_ NPs**) as a yellow powder. FTIR (neat): ν(cm^−1^) = 3415, 3035, 2926, 2853,1740, 1642, 1569, 1448,1383, 1261, 1092, 844, 741, 451. ^1^H-NMR (400 MHz, DMSO): δ = 1.463 (m, 2H), 2.985 (t, 2H), 4.515 (t, 2H), 8.160–859 (m, 7H), 8.613 (dd, 1H), 9.109 (dd, 1H).

### 2.4. Characterization Techniques

^1^H-NMR spectra were recorded on a 400 MHz spectrometer (Bruker AVANCE III, Billerica, MA, USA) using TMSO as an internal standard and DMSO as a deuterated solvent. Fourier transformed infrared (FTIR) spectra were attained on a spectrophotometer (Perkin Elmer 16F PC, Perkin Elmer Inc., Waltham, MA, USA). The phase of silica NPs was evaluated by X-ray diffractometer (Rigaku MiniFlexII, Tokyo, Japan) with Cu Kα1 radiation (γ = 0.15416 nm). Surface morphology and particle size of silica samples were investigated via field emission scanning electron microscope (Lyra-3, Tescan, Czech Republic) with an accelerating voltage up to 30 kV. A dilute dispersion of each sample was dried on a stub with Cu-tape followed by Au-coating. An energy dispersive X-ray (EDX) silicon-drift detector (X-Max⁠N, Oxford Instruments, Oxford, UK) coupled with FESEM was engaged to determine the presence and ratio of elemental particles. Thermogravimetric analyses (TGA) were performed on TGA 1 STARe System (Mettler Toledo, Columbus, OH, US) under Ar atmosphere (flow rate 15 mL min^−1^) from 20 to 800 °C at a rate of 10 °C min^−1^. The BET surface area of materials was estimated by N_2_ adsorption-desorption using a Micromeritics (ASAP 2010) analyzer. The surface charge and zeta potential values of synthesized NH_2_@SiO_2_ NPs and Pyr-NH@SiO_2_ NPs were evaluated using the Zetasizer nano (ZEN3600, Malvern, UK).

### 2.5. Photoluminescence Study

To assess the practicality of our synthesized nanosensor (Pyr-NH@SiO_2_ NPs) for mercury ion detection, the sensing material was well-dispersed in deionized water using the probe sonicator (UP400st). Photoluminescence (PL) spectra of as-synthesized Pyr-NH@SiO_2_ NPs were recorded by spectrofluorometer (FP-8500, JASCO) at an excitation wavelength of 340 nm by adjusting the bandwidth to 5 nm. All the measurements were recorded between 350–550 nm at ambient conditions and measured PL intensity at 398 nm. The sensing properties of Pyr-NH@SiO_2_ NPs (20 ppm) were recorded by the successive increase in Hg^2+^ ion concentration within the range from 0 to 50 ppm. Finally, the selectivity of Pyr-NH@SiO_2_ NPs against Hg^2+^ ions was examined in the presence of ubiquitous ions (Na^+^, K^+^, Ca^2+^, Mg^2+^, Ba^2+^, Ag^+^, and seawater samples). The total salinity of the seawater sample was ~36.03 g L^−1^. The detailed procedures for preparation of stock solutions, diluted solutions, and mixing of the fluorophore with quenchers are provided in the electronic supplementary file.

## 3. Results and Discussion

### 3.1. Synthesis Chemistry of Pyr-NH@SiO_2_ NPs

**Scheme 1:**
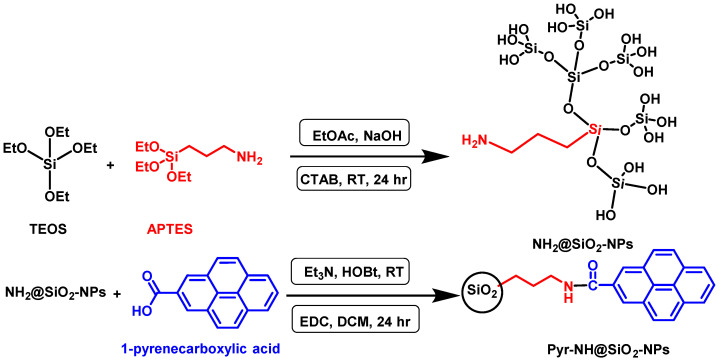
The synthesis scheme of pyrene attached silica nanoparticles (Pyr-NH@SiO_2_ NPs).

There are various well-documented methods to synthesize silica nanoparticles [31,32]. Among these methods, Stöber’s method is considered the most simple and efficient in terms of reaction conditions and high experimental yield [29,33]. The same method was adopted here to prepare mono-dispersed spherical silica nanoparticles by the hydrolysis of TEOS followed by a condensation reaction using ethyl acetate in the presence of sodium hydroxide and CTAB surfactant (Scheme 1). The surface of silica NPs was amino-functionalized to achieve NH_2_@SiO_2_ NPs by utilizing APTES under the same reaction conditions. In the second step, as-synthesized NH_2_@SiO_2_ NPs were subjected to amidation with 1-pyrenecarboxylic acid by the addition of hydroxybenzotriazole and 1-ethyl-3-(3-dimethylaminopropyl)carbodiimide hydrochloride as peptide coupling agents (Scheme 1) [34]. This coupling reaction was also performed by another route where 1-pyrenecarboxylic acid was first converted into an acid chloride using thionyl chloride. The excessive thionyl chloride was removed under reduced pressure or bubbling nitrogen gas in a fume-hood and then the acid chloride was allowed to react directly with NH_2_@SiO_2_ NPs in anhydrous chloroform [35]. The final product (Pyr-NH@SiO_2_ NPs) was extracted with ethyl acetate and washed the organic layer with a saturated solution of sodium bicarbonate to remove excessive or unreacted 1-pyrenecarboxylic acid (Figure S1, ESI).

The final product (Pyr-NH@SiO_2_ NPs) was synthesized using 1.21 mmol (0.300 g) of 1-pyrenecarboxylic acid and 0.200 g of NH_2_@SiO_2_ NPs. Therefore, the loading of 1-pyrenecarboxylic acid on NH_2_@SiO_2_ NPs was assessed via PL measurements for a series of concentrations of 1-pyrenecarboxylic acid ranging from 0.75 to 1.25 mmol, as shown in Figure S2 (ESI). Linear regression was obtained between the intensity of fluorescence and concentrations of 1-pyrenecarboxylic acid. The fluorescence of Pyr-NH@SiO_2_ NPs was treated as unknown. From the straight-line equation (R^2^ = 0.995), it has been estimated that 6.050 mmol of 1-pyrenecarboxylic acid per gram of silica are loaded up to 5.250 mmol per gram of silica.

### 3.2. Structural and Morphological Analyses

The chemical structures of NH_2_@SiO_2_ NPs and Pyr-NH@SiO_2_ NPs were investigated by ^1^H-NMR (Figure S3, ESI). The amino-functionalized silica nanoparticles (NH_2_@SiO_2_ NPs) are comprised of three different methylene (–CH_2_–) protons. The characteristic peak of central methylene protons in the propyl chain (–CH_2_–CH_2_–CH_2_–) appeared at δ = 1.24 ppm, methylene protons adjacent to Si–O (–CH_2_–Si–O) emerged at δ = 2.33 ppm, while the methylene protons near terminal amine (–NH_2_–CH_2_–) were found at δ = 2.67 ppm (Figure S3a). Similarly, the chemical structure of the final product (Pyr-NH@SiO_2_ NPs) was ascertained from ^1^H-NMR, where the methylene protons of the amino-propyl component showed significant downfield shifts. The methylene protons that existed adjacent to amide bond (Pyr–NH–CH_2_–) appeared at δ = 4.51 ppm as compared to δ = 2.67 ppm, CH_2_ near Si–O (–CH_2_–Si–O) shifted to δ = 2.98 ppm, while the central CH_2_ group in the propyl chain (–CH_2_–CH_2_–CH_2_–) moved slightly to δ = 1.47 ppm from δ = 1.24 ppm. The aromatic protons of the pyrene ring were found between δ = 8.16 ppm and δ = 9.11 ppm (Figure S3b).

The functional groups, stretching, and bending vibrations of NH_2_@SiO_2_ NPs and Pyr-NH@SiO_2_ NPs were investigated by FTIR spectroscopy (Figure S4, ESI). The FTIR spectrum of NH_2_@SiO_2_ NPs showed a broad band at 3444 cm^−1^ for N–H stretch that has been found overlapped with hydroxyl (–OH) of silica core or water adsorbed on the surface of as-synthesized material. However, the broad bands of N–H bending could be seen clearly at 1643 cm^−1^. The aliphatic nature of molecule for the aminopropyl part is fully supported by the stretching bands observed at 2922 cm^−1^ and 2852 cm^−1^ along with bending vibration bands at 1553 cm^−1^ and 1471 cm^−1^. The symmetric and antisymmetric vibration modes of Si–O–Si were appeared at 785 cm^−1^ and 1130 cm^−1^, respectively, and its bending vibration was found at 451 cm^−1^. The C–N stretching band was observed at 1056 cm^−1^ which is strongly overlapped with strong bands of silanol groups and Si–O–Si vibrations (Figure S4a) [36]. The FTIR spectrum of Pyr-NH@SiO_2_ NPs (final product) showed additional absorption bands for aromatic ring stretch at 3035 cm^−1^ and carbonyl group (C=O) stretching bands at 1740 cm^−1^ (Figure S4b) along with the characteristic peaks emerged for NH_2_@SiO_2_ NPs. The ^1^H-NMR and FTIR results confirm the formation of pyrene attached silica nanoparticles.

**Figure 1:**
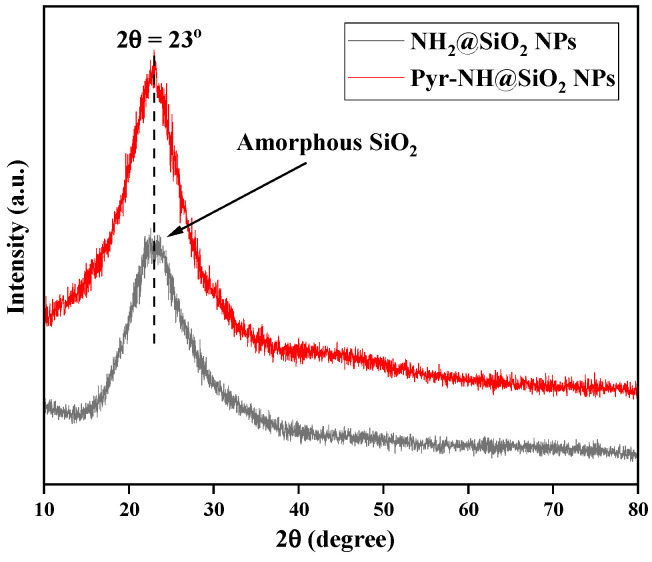
XRD patterns of (**–**) NH_2_@SiO_2_ NPs and (**–**) Pyr-NH@SiO_2_ NPs.

The crystal structure, phase, and purity of as-synthesized NH_2_@SiO_2_ NPs and Pyr-NH@SiO_2_ NPs were investigated by X-ray diffraction (XRD) analysis. A broad diffraction peak observed at 2θ position of ~23° (JCPDS No., 00-001-0649) confirmed the amorphous nature of silica nanoparticles (Figure 1) [37,38]. However, no extra peaks were detected, indicating the absence of any other crystalline phases in the synthesized materials. The products were thoroughly washed to remove the surfactant, unwanted coupling reagents, and unreacted 1-pyrenecarboxylic acid to avoid impurities.

**Figure 2:**
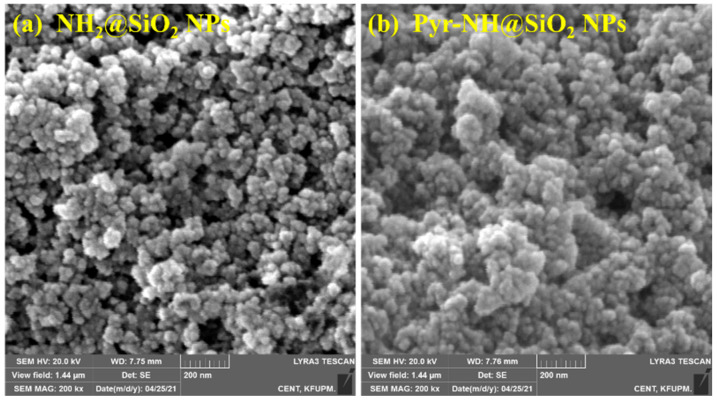
High-resolution FESEM images of (**a**) NH_2_@SiO_2_ NPs and (**b**) Pyr-NH@SiO_2_ NPs.

The surface morphology and particle size of as-synthesized silica and pyrene attached silica NPs were examined via field emission scanning electron microscopy (FESEM). Figure 2 (high-resolution) and Figure S5 (low-resolution) represent FESEM images of (a) NH_2_@SiO_2_ NPs and (b) Pyr-NH@SiO_2_ NPs, respectively. It seems that the spherical-shaped silica particles are well-dispersed, homogeneous in size, and uniformly distributed over the surface. The average size of NH_2_@SiO_2_ NPs and Pyr-NH@SiO_2_ NPs are ~35 and ~40 nm, respectively. The comparison indicates that the Pyr-NH@SiO_2_ NPs are more compact compared to NH_2_@SiO_2_ NPs, which may be due to aggregation occurring by π–π interactions between the organic moieties. Moreover, there is no significant change in the average size of silica NPs upon attachment with pyrene components. The elemental composition was evaluated via energy-dispersive X-ray spectroscopy (EDS) for the selected area of micrograph. The elemental mapping of Pyr-NH@SiO_2_ NPs signifies the presence of Si, O, C, and N atoms in the investigated sample (Figure S6, ESI). The elemental maps exhibit a homogenous distribution of all components.

**Figure 3:**
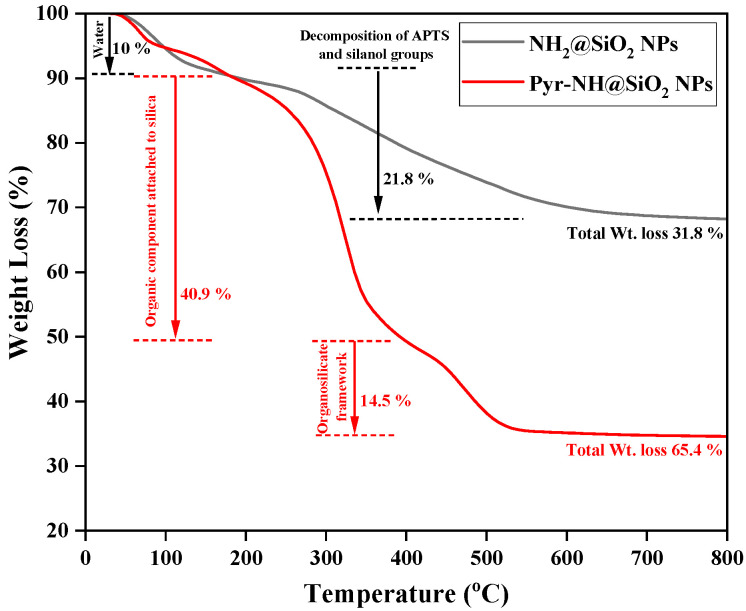
Thermogravimetric curves of (**–**) NH_2_@SiO_2_ NPs and (**–**) Pyr-NH@SiO_2_ NPs show the thermal decomposition of organic components and organosilicate frameworks attached to silica nanoparticles.

Considering the attachment of organic components with silica NPs, the thermal stability of amino-functionalized silica (NH_2_@SiO_2_ NPs) and pyrene-functionalized silica (Pyr-NH@SiO_2_ NPs) was investigated from room temperature to 800 °C (Figure 3). The first weight loss (~10%) was observed up to 160 °C due to moisture and water molecules that are physically adsorbed on the surface of silica NPs. The weight loss (~21.8%) detected in the region from 160 °C to 800 °C for NH_2_@SiO_2_ NPs is mainly due to the thermal decomposition of 3-aminopropyl and silanol groups [39], which confirm the chemical attachment of 3-aminopropyl groups with silica NPs. However, in the case of Pyr-NH@SiO_2_ NPs, the weight loss was observed in two consecutive steps. In the first step, the weight loss (40.9%) between 160 °C to 400 °C is attributed to the organic components attached to silica while the weight loss (14.5%) between 400 °C to 600 °C is mainly due to the thermal decomposition of organosilicate frameworks (Si–C, C–N, and C–C bonds) [28,40]. The observed weight losses also confirm the attachment of pyrene dye with silica NPs. The results indicate that the developed sensor having Pyr-NH@SiO_2_ NPs is thermally stable enough (up to 200 °C) for practical applications.

**Figure 4:**
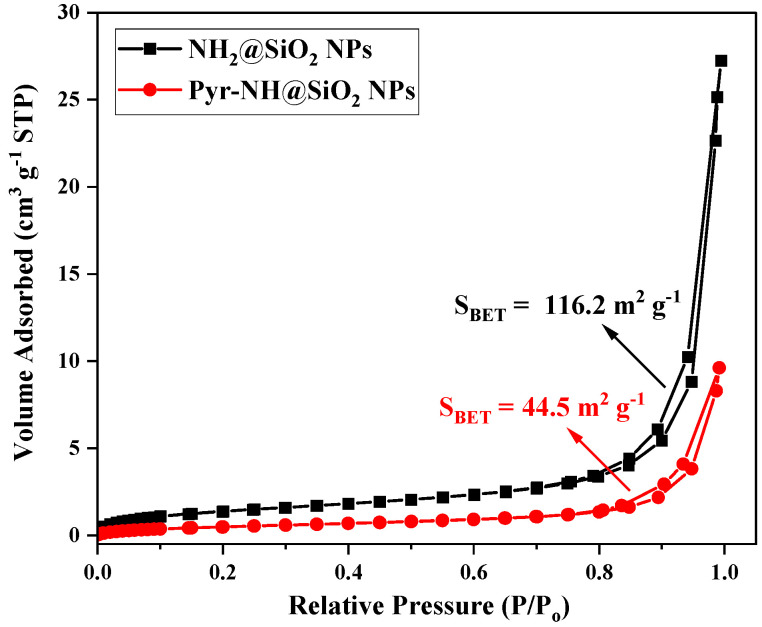
Nitrogen adsorption-desorption isotherms of (**▪**) NH_2_@SiO_2_ NPs and (●) Pyr-NH@SiO_2_ NPs.

The surface area and pore structure of NH_2_@SiO_2_ NPs and Pyr-NH@SiO_2_ NPs were assessed by nitrogen adsorption-desorption isotherms. The curves (Figure 4) represent type II isotherms at high relative pressure, which suggests the formation of macroporous silica materials having uniform size distributions [11]. The obtained parameters, such as BET surface area (S_BET_), total pore volume (V), and average pore diameter (D_BJH_), are summarized in Table 1. The comparison indicates that the BET surface area of NH_2_@SiO_2_ NPs (116.2 m^2^ g^−1^) decreases 2.61 times as compared to Pyr-NH@SiO_2_ NPs (44.5 m^2^ g^−1^). Moreover, the values of Barrett-Joyner-Halenda (BJH) pore sizes for synthesized NH_2_@SiO_2_ NPs and Pyr-NH@SiO_2_ NPs were found to be 30.8 nm and 25.3 nm, respectively. This decrease in surface area, pore size, and pore volume of Pyr-NH@SiO_2_ NPs signifies the blocking of pore surfaces and channel walls, which also confirms the presence of a fluorescence indicator (pyrene) on the inner surface of silica NPs.

The surface charge and zeta potential values of as-synthesized NPs were estimated in deionized water by the dynamic light scattering (DLS) technique. The NH_2_@SiO_2_ NPs and Pyr-NH@SiO_2_ NPs were found positively charged, having zeta potential values 1.69 mV and 38.0 mV, respectively (Figure S7). The comparison indicates that the Pyr-NH@SiO_2_ NPs are more stable in water after modification with 1-pyrene-carboxylic acid due to the formation of stable hydrogen bonding with water molecules in the presence of N–H and C=O groups [11]. This indicates that Pyr-NH@SiO_2_ NPs can be successfully deployed as a chemosensor in aqueous environments. The average particle size distribution of Pyr-NH@SiO_2_ NPs was found to be 62 nm (Figure S8), which agrees with the SEM results.

### 3.3. Fluorescent and Sensing Properties of Pyr-NH@SiO_2_ NPs

**Figure 5:**
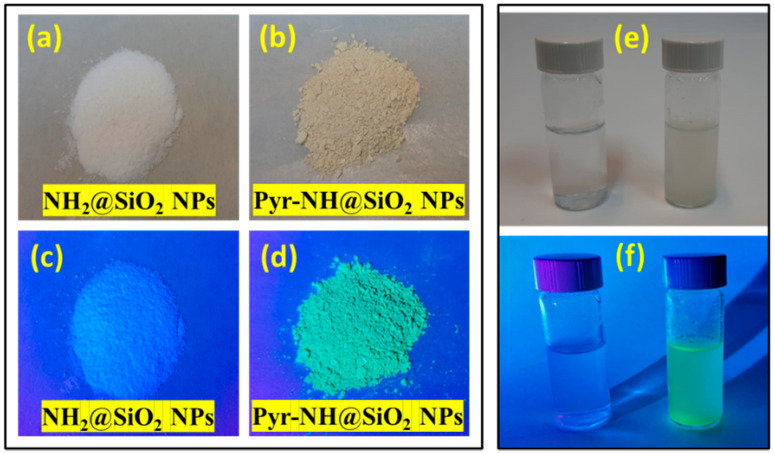
Pictograms of as-synthesized powdered samples under (**a**,**b**) normal light and (**c**,**d**) UV light illumination. The fluorescence properties of NH_2_@SiO_2_ NPs and Pyr-NH@SiO_2_ NPs in deionized water under (**e**) normal light and (**f**) UV light.

The luminescent properties of as-synthesized powdered samples (NH_2_@SiO_2_ NPs and Pyr-NH@SiO_2_ NPs) were examined under Figure 5a,b normal light as well as (Figure 5c,d) UV-light illumination. The pictogram of Pyr-NH@SiO_2_ NPs (Figure 5d) exhibits luminescence characteristics under UV-light (λ ~395 nm) due to the attachment of fluorescent pyrene with silica NPs. Similarly, the luminescent properties of NH_2_@SiO_2_ NPs and Pyr-NH@SiO_2_ NPs dispersions were also investigated in deionized water under (Figure 5e) normal light and (Figure 5f) UV light. It was observed that the synthesized Pyr-NH@SiO_2_ NPs remained dispersed in the aqueous phase and produced bright green fluorescence emission under UV-light illumination (Figure 5f). This also indicates the chemical and fluorescence stability of Pyr-NH@SiO_2_ NPs in the aqueous environment.

**Figure 6:**
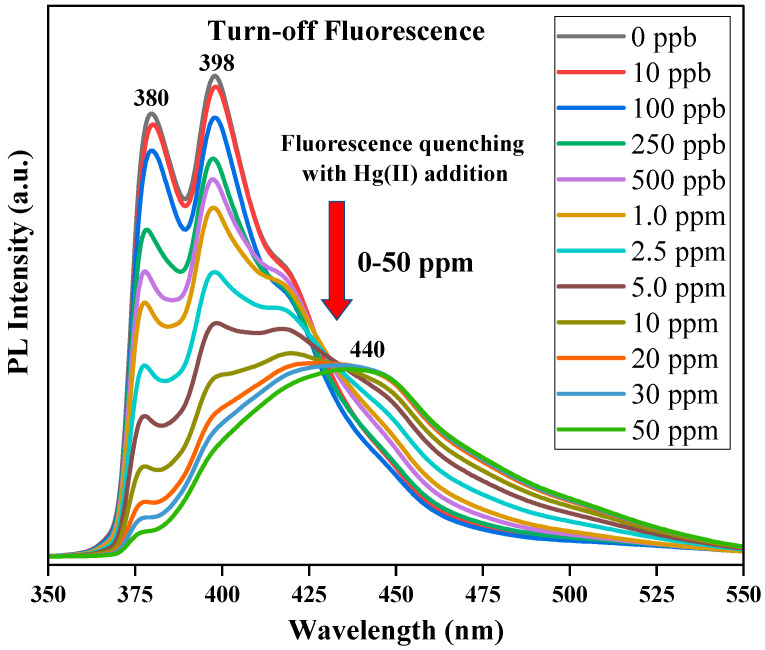
PL emission spectra of Pyr-NH@SiO_2_ NPs (20 ppm) before and after exposure to Hg^2+^ ions in the concentration ranged from 0–50 ppm (λ_ex_ = 340 nm).

Prior to fluorescence studies, UV-Visible spectra of Py-NH@SiO_2_ NPs were recorded at various concentrations of 20, 40, and 100 ppm (Figure S9). The spectra show two absorption maxima at 275 and 340 nm. These absorption maxima will be useful to excite the synthesized material for photoluminescence (PL) studies. The fluorescent properties of Pyr-NH@SiO_2_ NPs were investigated to check their feasibility for mercury ions detection. Figure 6 represents photoluminescence (PL) emission spectra of Pyr-NH@SiO_2_ NPs before and after exposure to Hg^2+^ ions. The changes in fluorescent properties were examined on the basis of peak shift and emission intensity. The PL spectrum of Pyr-NH@SiO_2_ NPs (20 ppm) exhibits two distinct vibronic bands observed at 380 and 398 nm that correspond to π → π* transitions in the pyrene molecule, which are cumulatively denoted as the monomeric emission [41,42]. Pyr-NH@SiO_2_ NPs were further exposed to the known concentrations of Hg^2+^ ions (0–50 ppm), and PL intensity was measured from the emission spectra at 398 nm. It was observed that the fluorescence emission intensity of pyrene gradually decreases when Hg^2+^ concentration increases from 0 to 50 ppm. This gradual decline in fluorescence intensity can be attributed to the possible complexation of Hg^2+^ ions with fluorescent pyrene molecules to form a stable mercury-pyrene complex, which triggers the turn-off (quenching) mechanism. The quenching phenomenon can be divided into two types: static and dynamic quenching. Static quenching occurs through the formation of a complex while dynamic quenching proceeds via the random collision between emitter and quencher. However, in both cases, the electron or energy transfer happens from the emitter to quencher. This transfer rate can be measured quantitatively by the Stern-Volmer equation [43]. In the case of dynamic quenching, the quenching rate can be measured from the slope of the Stern–Volmer plot and lifetime measurements. While the static quenching results due to the complexation of analytes and receptors that led to the creation of new absorption, relaxation, or energy transfer processes that favor nonradiative decay or through a new exciton/emission behavior. Therefore, the equilibrium constant of static quenching (K_sv_) can be calculated from the Stern-Volmer plot and considered as the binding constant for the quencher-acceptor system [44]. The Stern–Volmer equation is represented in Equation (1),

(1) $$\frac{\;F{}^{\circ}}{F}=1+K_{SV}\left[Q\right]$$

where **F°** is the fluorescence intensity of sensing material without quencher, **F** is the fluorescence intensity of the sensing material in the presence of Hg^2+^ ions, Q is the concentration of quencher, and **K_sv_** is the Stern–Volmer constant that explains the quantitative measure of quenching [45,46]. A straight-line plot was obtained from **F**°**/F** versus concentration that indicates the static quenching where the complex is formed between the sensor and quencher. The quenching mechanism is consistent with the similar complex reported in the literature [47]. The fluorescence intensity of Pyr-NH@SiO_2_ NPs in the presence of Hg^2+^ ions is quenched. It was observed that the fluorescence decreased with time and became constant after a certain time. The equilibrium constant (**K_sv_**) of Pyr-NH@SiO_2_ NPs with Hg^2+^ ions was found to be 0.114 × 10^6^ M^−1^ (Figure S10). Moreover, we observed a hypsochromic shift (blue shift) in the peak positions (380, 398 nm) and emission of excimer (~440 nm) upon Hg^2+^ ion addition. The fluorescence quenching, peak shifting, and excimer emission are attributed to the possible photoinduced electron transfer to pyrene molecule and the formation of a stable Hg-pyrene complex with emitting chromophore [47,48]. It was observed that the presence of only a small amount of Hg^2+^ ions (ppb level) reduce the fluorescence intensity of pyrene up to a great extent (Figure 6). Therefore, the limit of detection (LOD) was estimated for Hg^2+^ ion sensing, which is 3.3 times higher than the standard deviation of measurement [49]. The results show that the developed fluorescent sensor can reliably quantify Hg^2+^ ions ≥ 10 ppb (0.05 µM) and the best linear working range is 10–500 ppb (0.05–2.50 µM) in aqueous environments.

**Figure 7:**
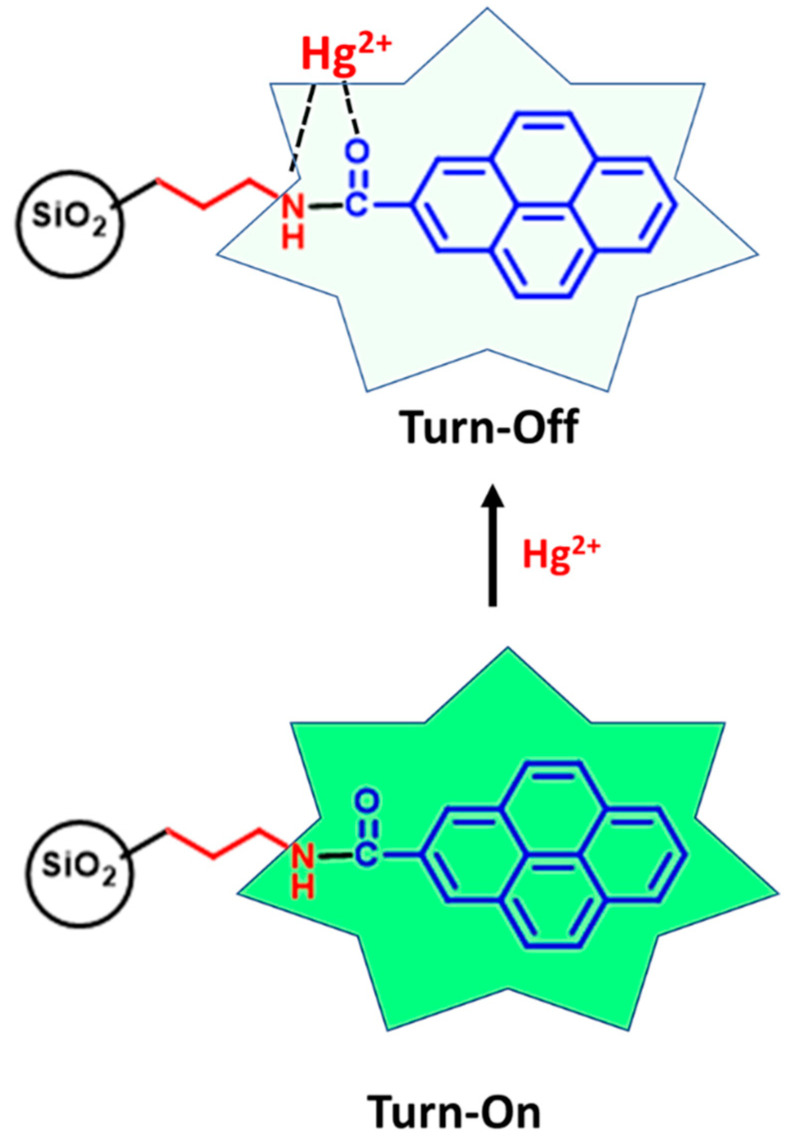
A possible mechanism for interaction of Hg^2+^ ions with Pyr-NH@SiO_2_ NPs.

The interaction of pyrene-based complexes can be predicted for mercury ions from the literature. For instance, Wei et al. [50] has shown that Hg^2+^ ions can interact through the O=C–NH group and result in the formation of a coordination complex. On this basis, a possible mechanism has been proposed for the interaction of Hg^2+^ ions with Pyr-NH@SiO_2_ NPs (Figure 7). Similar to the literature, the developed fluorescent materials contain the amide group, which might be responsible for forming the coordination complex that results in quenching the fluorescence intensity of Pyr-NH@SiO_2_ NPs. 

The binding stoichiometry of Pyr-NH@SiO_2_ NPs and Hg^2+^ ions was estimated via Job’s plot analysis. In this study, the total concentration of Pyr-NH@SiO_2_ NPs and Hg^+2^ ions were maintained at 5 µM and their mole fractions varied between 0 and 1. The plot of fluorescence intensity versus the mole fractions of Pyr-NH@SiO_2_ NPs/Hg^2+^ is shown in Figure S11 (ESI). The plot analysis indicates that the fluorescence intensity goes to a maximum value at a mole fraction of ~0.5. Therefore, the Pyr-NH@SiO_2_ NPs and Hg^2+^ represent the binding stoichiometry of 1:1, which is the most suitable binding mode between them [11], as demonstrated in Figure 7.

**Figure 8:**
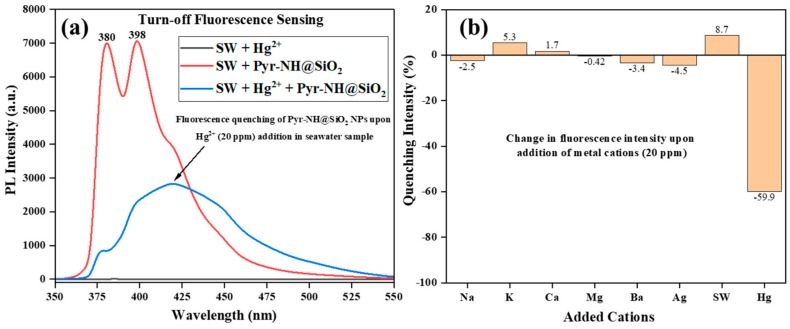
(**a**) PL emission spectra of real seawater (SW) samples which demonstrate the fluorescence quenching of Pyr-NH@SiO_2_ NPs (20 ppm) upon addition of Hg^2+^ ions (20 ppm); λ_ex_ = 340 nm. (**b**) Selectivity of Pyr-NH@SiO_2_ NPs against Hg^2+^ ions upon addition of major cations of seawater, such as Na^+^, K^+^, Ca^2+^, Mg^2+^, Ba^2+^, and Ag^+^ ions. All cations were used in the form of their chloride salts.

The developed sensor was also tested for recognition of Hg^2+^ ions present in a real seawater sample. Figure 8a demonstrates that the fluorescence intensity of Pyr-NH@SiO_2_ NPs (20 ppm) quenches ~60% with the addition of spiked Hg^2+^ ions (20 ppm). This indicates the effective recognition of Hg^2+^ ions in the presence of competitive metal cations in the seawater sample. In order to investigate the selectivity of as-synthesized Pyr-NH@SiO_2_ NPs sensors, the major cations of seawater, such as Na^+^, K^+^, Ca^2+^, Mg^2+^, Ba^2+^, and Ag^+^ ions, were individually analyzed with Pyr-NH@SiO_2_ NPs. It is estimated that the most suitable binding mode of Hg^2+^ ions with pyrene is 1:1 stoichiometry. Therefore, the optimum concentration for each metal cation was kept at 20 ppm against Pyr-NH@SiO_2_ NPs (20 ppm) and fluorescence intensity was measured at 398 nm. Figure 8b demonstrates that there is a slight change in the fluorescence intensity of Pyr-NH@SiO_2_ NPs upon the addition of each competitive cation. However, there is a drastic quenching (~60%) of fluorescence intensity upon Hg^2+^ addition due to the formation of a stable mercury-pyrene complex, which demonstrates the reliability as well as selectivity of the developed sensor for seawater samples. The developed chemosensor was cross verified with the help of the USEPA modified method 7473 on Nippon MA-3000 direct mercury analyzer. All three concentrations (25, 50, and 100 ppb) of Hg^2+^ ions were precisely matched with the mercury concentrations utilized in the Pyr-NH@SiO_2_ NPs chemosensor.

The analytical detection capability of the developed chemosensor is comparable or better with the other reported famous analytical methods for sensing Hg^2+^ ions (Table 2). The electrochemical sensors were found to be more sensitive than the chemosensors. However, it depends upon the type of the electrode materials [51]. Moreover, the selectivity of electrochemical sensors remains a critical challenge, and small changes in the sensing environment can bring a drastic alteration in the results. Similarly, Senapati et al. [52] developed a SERS probe for Hg^2+^ detection, which has a limit of detection up to 5 ppb. A colorimetric sensor based on dithizone has also shown the limit of detection for Hg^2+^ ions up to 10 ppb [53]. The comparison indicates that the designed Pyr-NH@SiO_2_ NPs have excellent potential to be used for Hg^2+^ sensing at trace level, and its simple synthesis protocol makes it advantageous over other reported sensors (Table 2).

## 4. Conclusions

Pyrene-attached silica nanoparticles (Pyr-NH@SiO_2_ NPs) were successfully synthesized by the chemical attachment of pyrene with amino-functionalized silica NPs using peptide coupling agents. The chemical structure of amino-functionalized pyrene and its covalent attachment with silica NPs was confirmed by ^1^H-NMR, FTIR, TGA, and BET results. The XRD results confirmed the amorphous nature of as-synthesized silica NPs, and their average particle size was found to be ~40 nm. Pyr-NH@SiO_2_ NPs are stable in the aqueous environment after modification with 1-pyrenecarboxylic acid due to the formation of stable hydrogen bonding with water molecules in the presence of N–H and C=O groups. The synthesized fluorescent particles have the ability to produce bright green emission under UV light. The fluorescence quenching, hypsochromic peak shifting (380, 398 nm), and excimer emission (~440 nm) upon the addition of Hg^2+^ ions are attributed to the photoinduced electron transfer to pyrene molecule and the formation of a stable Hg-pyrene complex with the emitting chromophore. The developed sensor can reliably and selectively recognize Hg^2+^ ions (LOD: 10 ppb) in the presence of ubiquitous metal cations and seawater samples. The fluorescent Pyr-NH@SiO_2_ NPs have great potential to design highly sensitive, selective, and portable opto-chemical mercury sensors for seawater applications.

## Figures and Tables

**Table 1:** Various parameters were obtained from N_2_ adsorption-desorption isotherms, such as BET surface area (S_BET_), total pore volume (V), and average pore diameter (D_BJH_).

Material	S_BET_/(m^2^ g^−1^)	V/(cm^3^ g^−1^)	D_BJH_/(nm)
NH_2_@SiO_2_ NPs	116.2	0.95	30.8
Pyr-NH@SiO_2_ NPs	44.5	0.33	25.3

**Table 2:** Comparisons of various fluorescent chemo-sensors for Hg^2+^ ions sensing.

Sr. #	Fluorescent Sensor	Limit of Detection (LOD)	Type of Sensor	Application	Selectivity	Ref.
1	Thioether-rich crown-based highly selective fluorescent sensor	50 ppb	Turn-on for Hg(II)	Living cell imaging of Hg(II)	Yes	[54]
2	Coumarin-based probe with an N′-acetylthioureido group	2 ppb	Turn-on	Not applied	Yes	[55]
3	Isocoumarin-based fluorescent probe of 2-amino-4-phenyl-6-(isocoumarin-3-yl)-3-cyanopyridine (APICP) synthesized from 3-acetyl isocoumarin and 2-benzylidene malononitrile	8.12 nM	Turn-off	Water	Yes	[56]
4	Fluorescent iron oxide NPs	3.650 nM	Turn-off	Mineral water, river water, and wastewater	Yes	[57]
5	Mercury green 1	10 ppb	Turn-on	Mercuric ions in living cells	Yes	[58]
6	Mercuryfluor-1	60 nM	Turn-on	Mercury levels in fish	Yes	[59]
7	Ru@UiO-66-NH_2_	53 nM	Turn-off	Hg^2+^ detection in lake water, tap water, and drink water	Yes	[60]
8	Acenaphtoquinoxaline	42 ppb	Turn-off	-	Yes	[61]
9	7-nitrobenzo-2-oxa-1,3-diazolyl (NBD) fluorophore and the thiophene ionophore	3.9 ppb	Turn-on	Hg^2+^ in drinking water, HepG2 cells, and Napa cabbage stems	Yes	[62]
10	Pyr-NH@SiO_2_ NPs	10 ppb	Turn-off	Seawater	Yes	This work

## Data Availability

The data presented in this study are available upon reasonable request from the corresponding author.

## Supplementary Materials

The following supporting information can be downloaded at: https://www.mdpi.com/article/10.3390/nano12081249/s1, Figure S1: The synthesis scheme (2^nd^ route) of Pyr-NH@SiO_2_ NPs without using coupling agents; Figure S2: Loading of 1-pyrenecarboxylic acid on amino-functionalized silica nanoparticles (NH_2_@SiO_2_ NPs). Figure S3: ^1^H-NMR spectra of (a) NH_2_@SiO_2_ NPs and (b) Pyr-NH@SiO_2_ NPs; Figure S4: FTIR spectra of (a) NH_2_@SiO_2_ NPs and (b) Pyr-NH@SiO_2_ NPs; Figure S5: Low-resolution FESEM images of (a) NH_2_@SiO_2_ NPs and (b) Pyr-NH@SiO_2_ NPs; Figure S6: Elemental maps of Pyr-NH@SiO_2_ NPs exhibit the presence of Si, O, C, and N atoms; Figure S7: Surface charge and zeta potential measurements of (a) NH_2_@SiO_2_ NPs and (b) Pyr-NH@SiO_2_ NPs; Figure S8: Size distribution of Pyr-NH@SiO_2_ NPs measured by DLS technique; Figure S9: UV-visible spectra of Py-NH@SiO_2_ NPs at various concentration of 20, 40, and 100 ppm; Figure S10: Stern–Volmer plot to measure equilibrium constant (K_sv_) of Pyr-NH@SiO_2_ NPs with Hg^2+^ ions (K_sv_ = 0.114 × 10^6^ M^−1^); Figure S11: Job’s plot for determining the binding stoichiometry of Pyr-NH@SiO_2_ NPs and Hg^+2^ ions. The total concentration of Pyr-NH@SiO_2_ NPs and Hg^+2^ ions was 5 µM. (λ_ex_ = 340 nm).
