# Supplementary material for: Cost-Effective and Selective Fluorescent Chemosensor (Pyr-NH@SiO2 NPs) for Mercury Detection in Seawater
Source: Nanomaterials (Basel). 2022 Apr 7;12(8):1249. doi: 10.3390/nano12081249 (PMC9024866; doi:10.3390/nano12081249)
Supplement: Supplementary file 1 [file nanomaterials-12-01249-s001.zip › nanomaterials-1615642-supplementary.pdf]

<u>Electronic Supplementary Information</u>

Cost-Effective and Selective Fluorescent Chemosensor ($Pyr-NH@SiO_2$ NPs) for Mercury Detection in Seawater

Shahid Ali [1,2,†], Muhammad Mansha [2,†], Nadeem Baig [3] and Safyan Akram Khan [1,2,*]

[1] Center of Research Excellence in Nanotechnology, King Fahd University of Petroleum and Minerals, Dhahran 31261, Saudi Arabia; s.ali@kfupm.edu.sa
[2] Interdisciplinary Research Center for Hydrogen and Energy Storage, King Fahd University of Petroleum and Minerals, Dhahran 31261, Saudi Arabia; manshachohan@kfupm.edu.sa
[3] Interdisciplinary Research Centre for Membranes and Water Security, King Fahd University of Petroleum and Minerals, Dhahran 31261, Saudi Arabia; nadeembaig@kfupm.edu.sa
* Correspondence: safyan@kfupm.edu.sa; Tel.: +966-13-860-7261
† These authors contributed equally to this work.

Preparation of various solutions/dispersions for photoluminescence study

- **Preparation of Hg^{2+} solutions**

A stock solution of Hg^{2+} (100 ppm, 100 mL) ions was prepared from mercury standard solution (1000 ppm, Sigma-Aldrich). Then, various dilutions (20 mL) of Hg^{2+} ions such as (100 ppm, 60 ppm, 40 ppm, 20 ppm, 10 ppm, 5 ppm, 2 ppm, 1000 ppb, 500 ppb, 200 ppb, 20 ppb, and 0 ppb) were prepared by applying dilution formula.

- **Preparation of Pyr-NH@SiO_2 NPs dispersions**

A stock dispersion of Pyr-NH@SiO_2 NPs (100 ppm, 100 mL) was prepared by dispersing 10.0 mg of fluorophore in deionized water (100 mL) using a probe sonicator (UP400st). Then, the second stock dispersion of Pyr-NH@SiO_2 NPs (40 ppm, 100 mL) was prepared by applying a dilution formula. The dispersion was well-dispersed using a probe sonicator before mixing with the quencher.

- **Mixing of Pyr-NH@SiO_2 NPs with Hg^{2+} ions**

Equal volumes of Pyr-NH@SiO_2 NPs (40 ppm) and Hg solutions (100 ppm, 60 ppm, 40 ppm, 20 ppm, 10 ppm, 5 ppm, 2 ppm, 1000 ppb, 500 ppb, 200 ppb, 20 ppb, and 0 ppb) were mixed in the bottles. The final concentration of Pyr-NH@SiO_2 NPs in each bottle was 20 ppm. While the final concentrations of Hg^{2+} ions were 50 ppm, 30 ppm, 20 ppm, 10 ppm, 5 ppm, 2.5 ppm, 1 ppm, 500 ppb, 250 ppb, 100 ppb, 10 ppb, and 0 ppb in the final dispersions.

- **Mixing of Pyr-NH@SiO_2 NPs with other metal cations**

The major cations of seawater such as Na^+, K^+, Ca^{2+}, Mg^{2+}, Ba^{2+}, and Ag^+ ions were selected for selectivity of mercury sensing. Therefore, the stock solution of each metal cation (100 ppm, 100 mL) was prepared from its standard solution (1000 ppm, Sigma-Aldrich). Then, 40 ppm of the diluted solution was prepared for each metal cation by applying the dilution formula. A similar protocol was adopted for the mixing of Pyr-NH@SiO_2 NPs (40 ppm) dispersion with metal cation solution (40 ppm) to get the final dispersion of 20 ppm each.

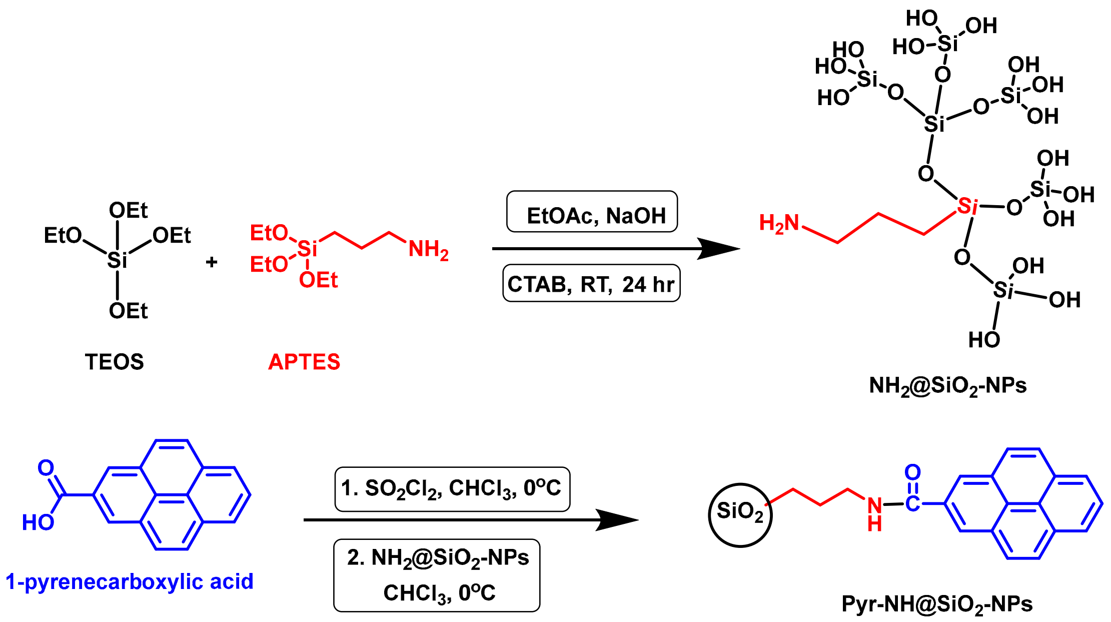

Figure S1: The synthesis scheme (2nd route) of Pyr-NH@SiO_2 NPs without using coupling agents.

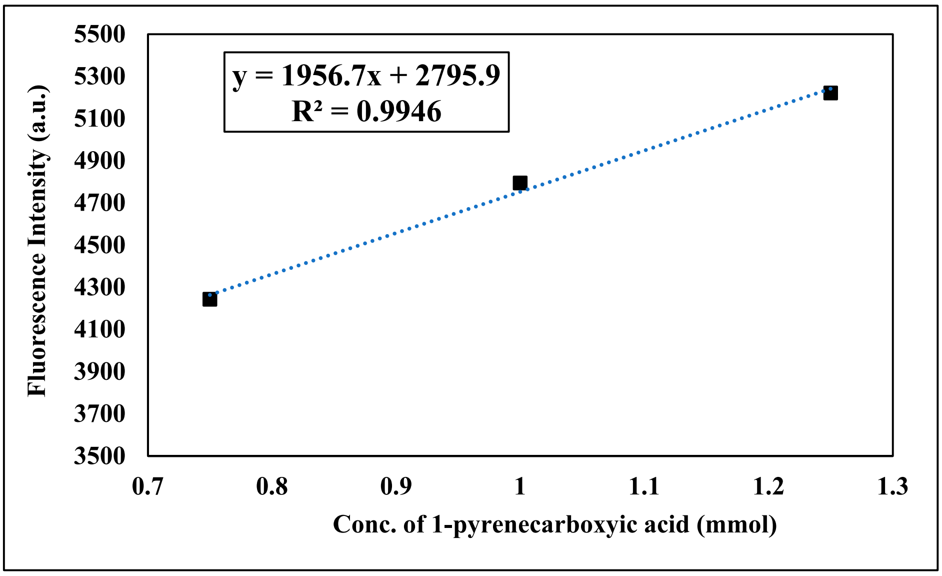

Figure S2: Loading of 1-pyrenecarboxylic acid on amino-functionalized silica nanoparticles (NH_2@SiO_2 NPs).

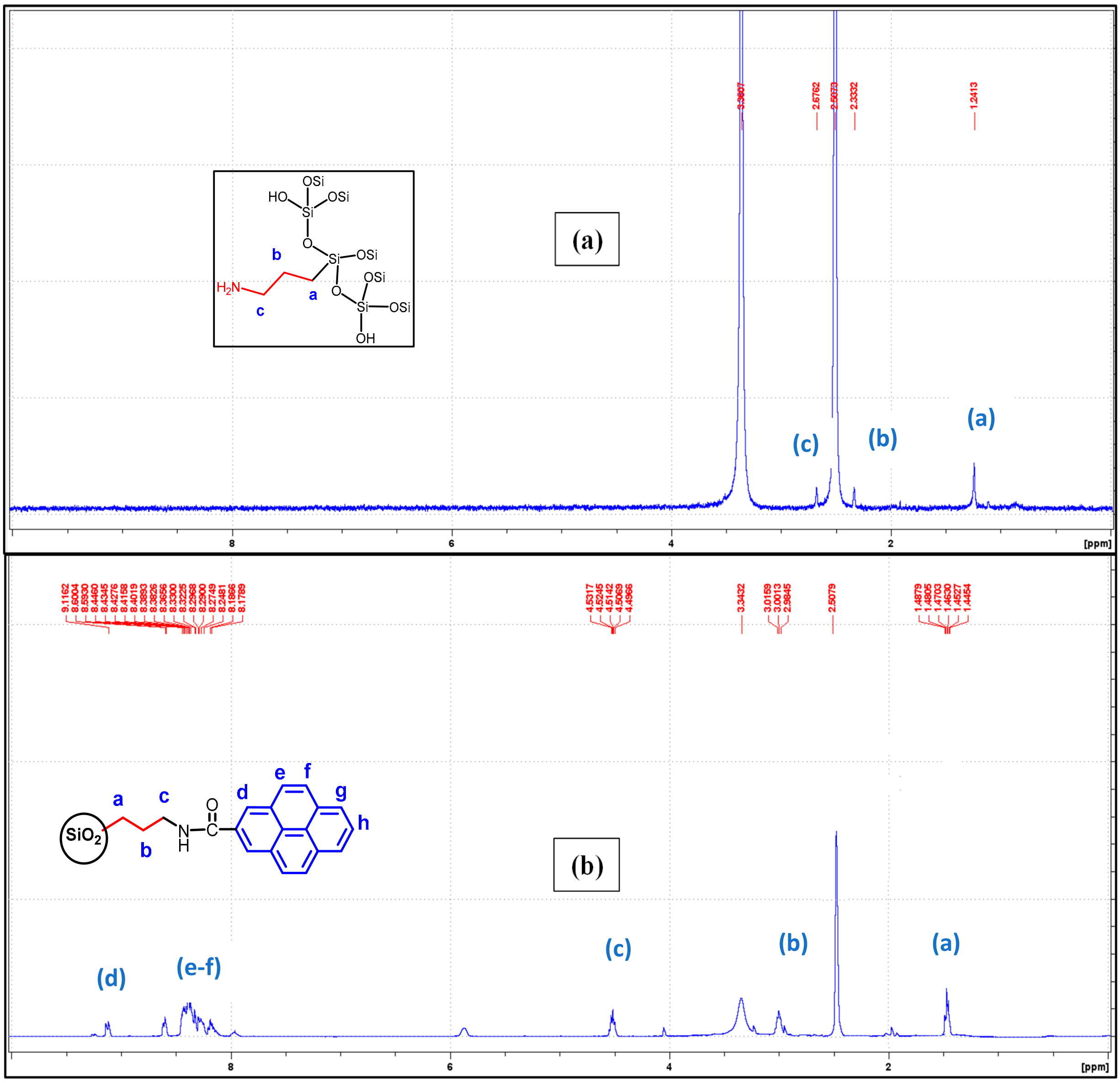

Figure S3: ^{1}H-NMR spectra of (a) $NH_2@SiO_2$ NPs and (b) Pyr-NH@SiO_2 NPs.

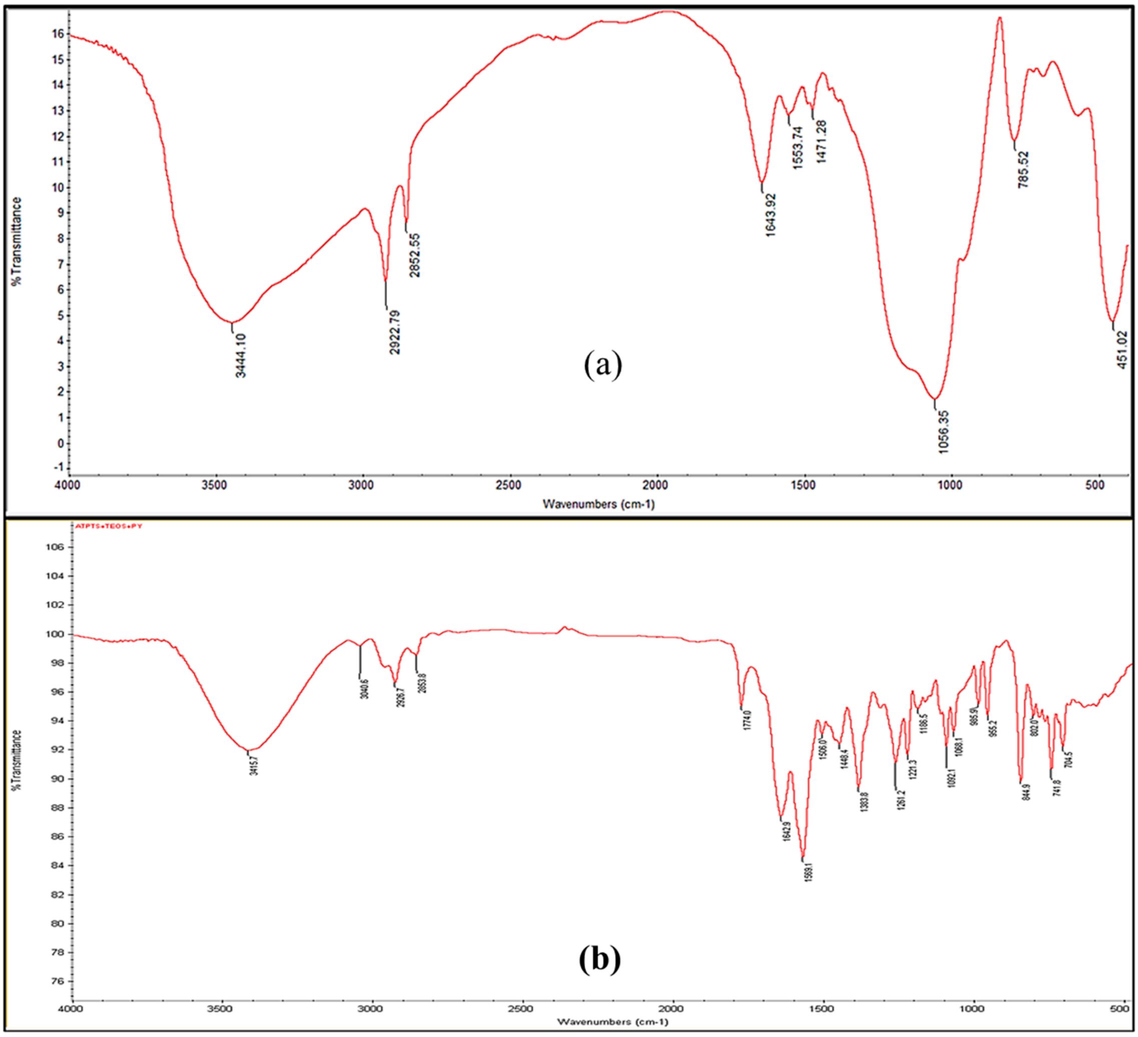

Figure S4: FTIR spectra of (a) $NH_2@SiO_2$ NPs and (b) Pyr-NH@SiO_2 NPs.

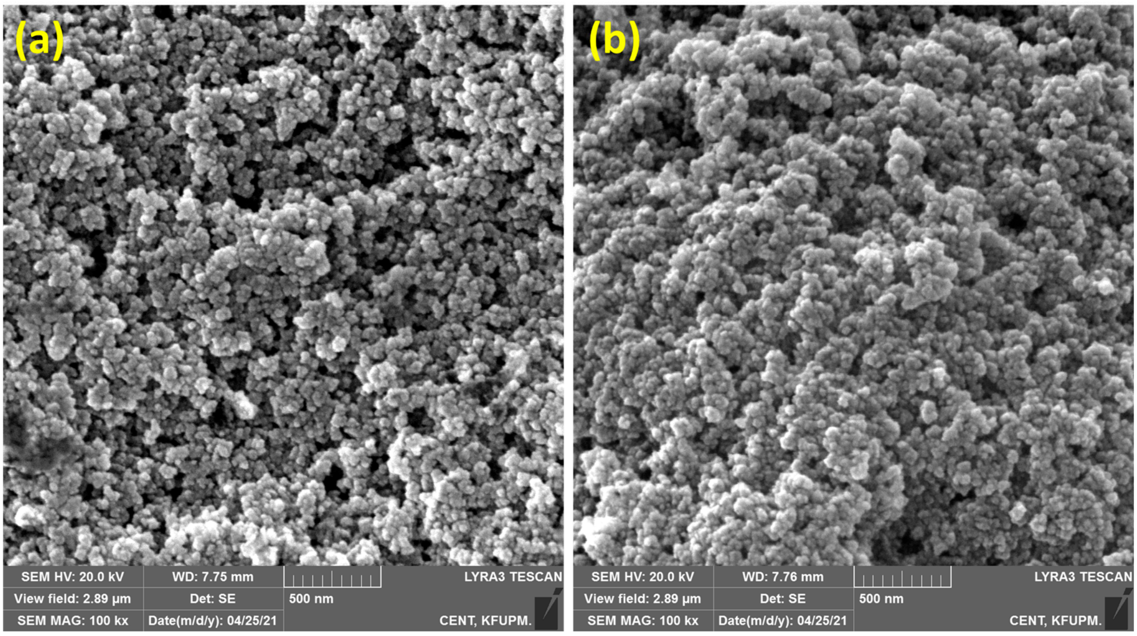

Figure S5: Low-resolution FESEM images of (a) NH_2@SiO_2 NPs and (b) Pyr-NH@SiO_2 NPs.

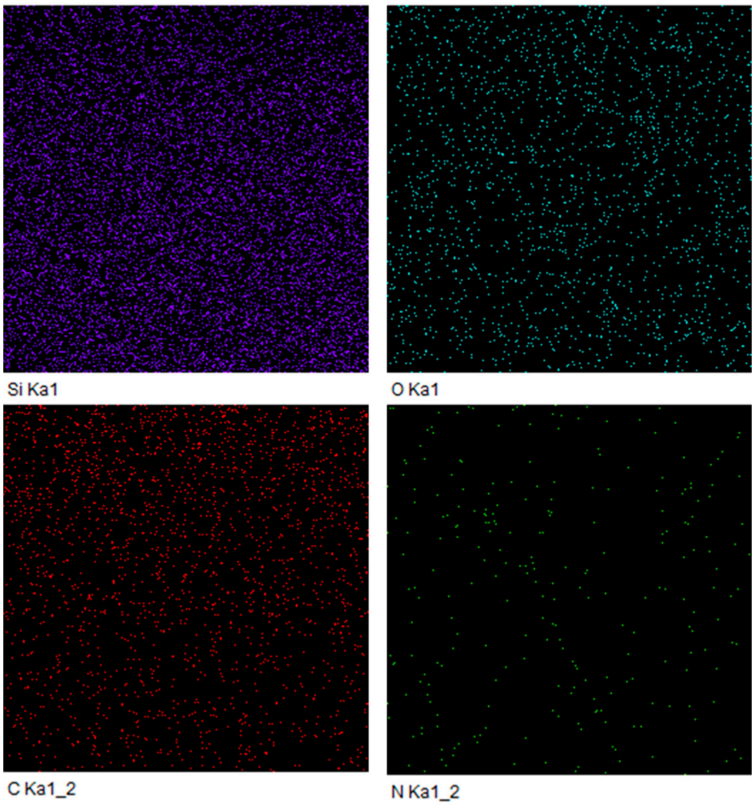

Figure S6. Elemental maps of Pyr-NH@SiO_2 NPs exhibit the presence of Si, O, C, and N atoms.

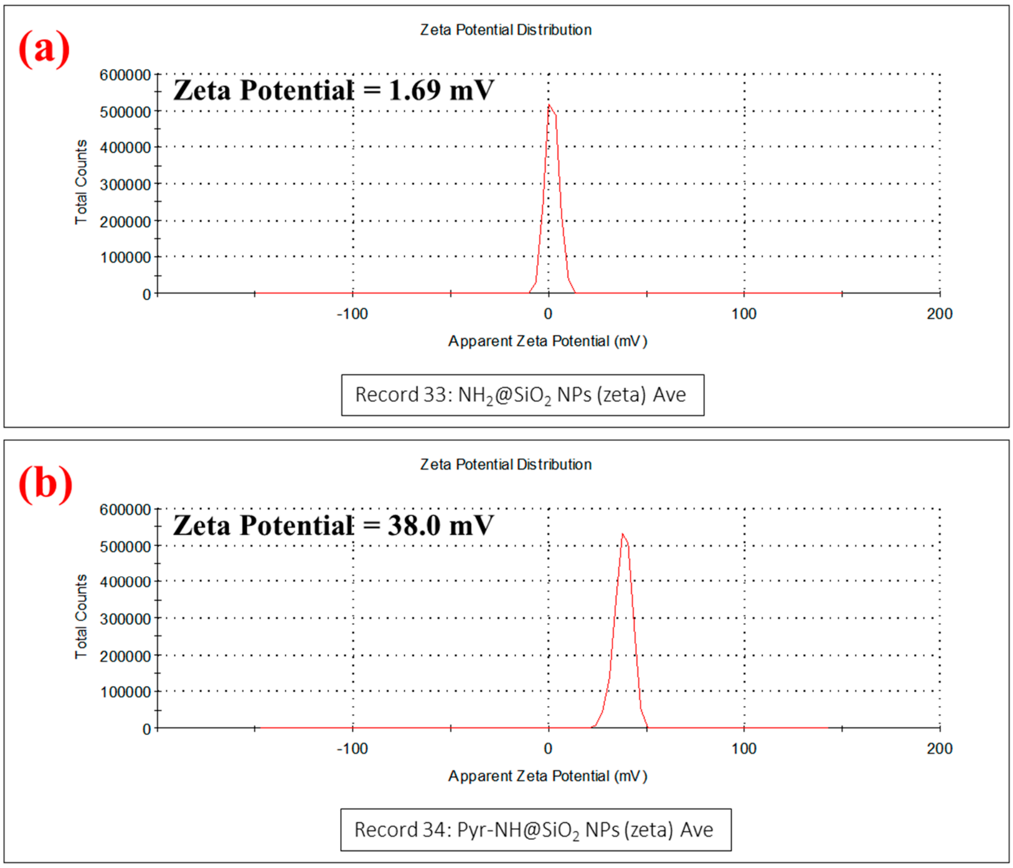

Figure S7: Surface charge and zeta potential measurements of (a) $NH_2@SiO_2$ NPs and (b) Pyr-$NH@SiO_2$ NPs.

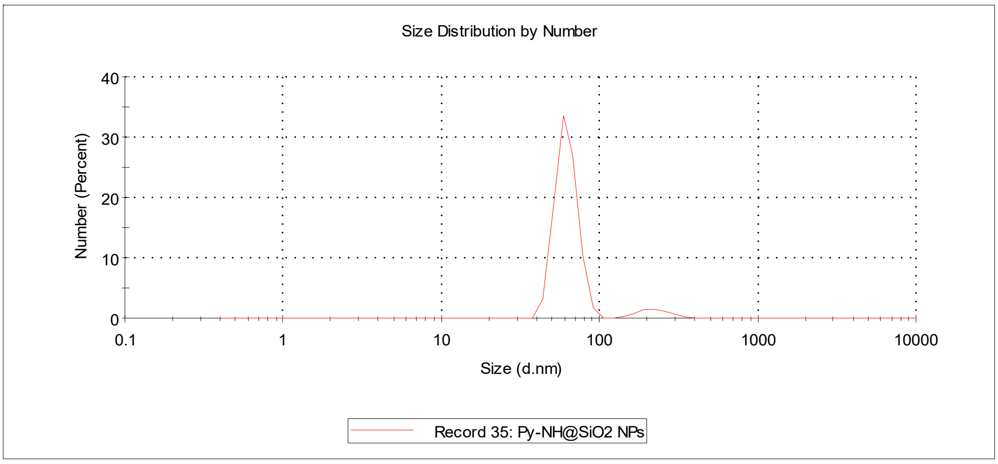

Figure S8: Size distribution of Pyr-$NH@SiO_2$ NPs measured by DLS technique.

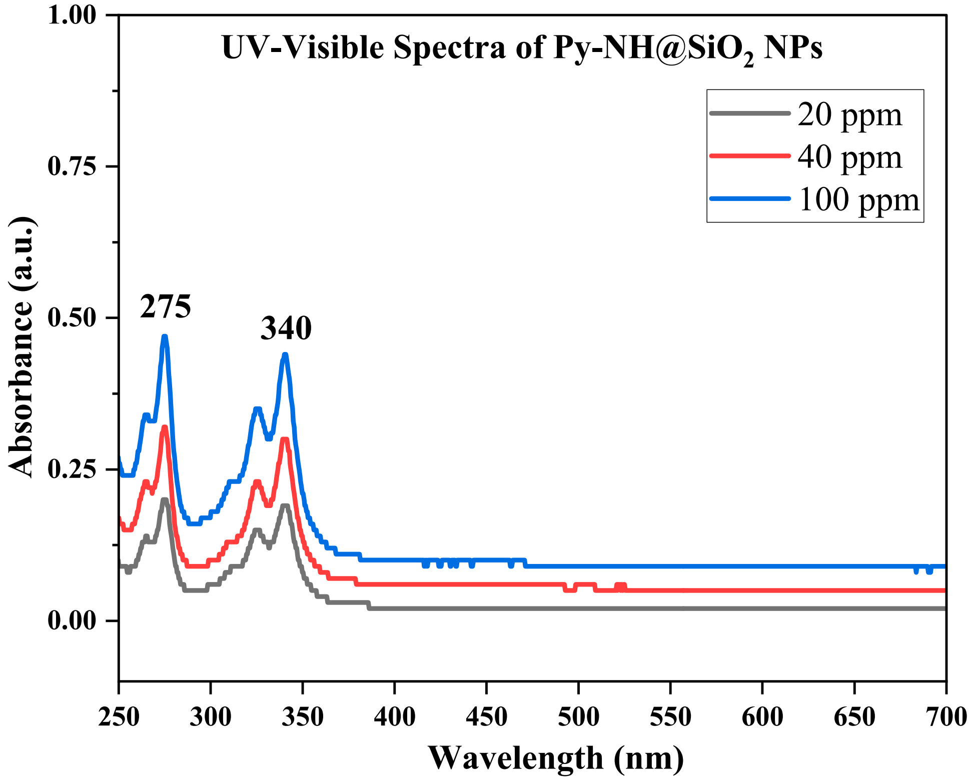

Figure S9: UV-visible spectra of Py-NH@SiO_2 NPs at various concentrations of 20, 40, and 100 ppm.

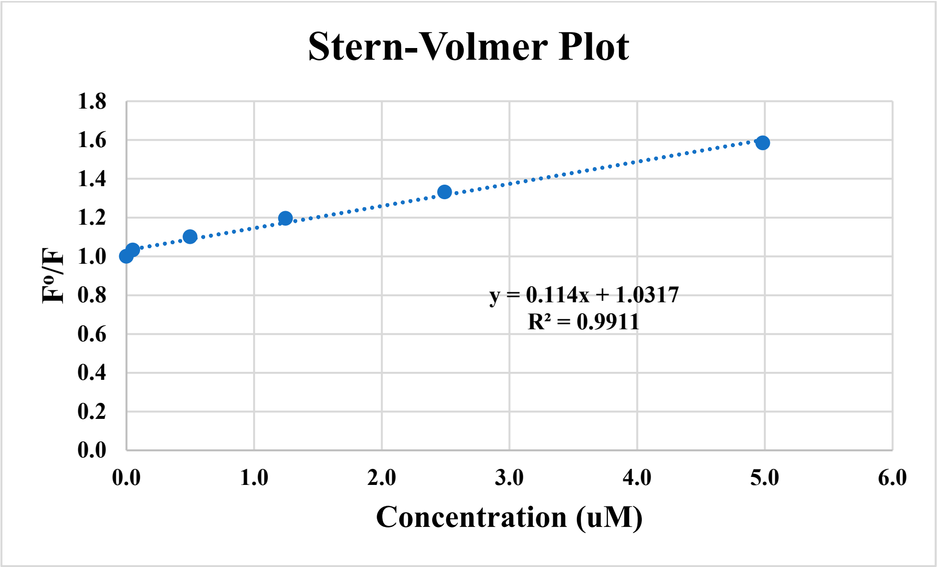

Figure S10: Stern-Volmer plot to measure equilibrium constant (K_{sv}) of Pyr-NH@SiO_2 NPs with Hg^{2+} ions ($K_{sv} = 0.114 \times 10^6\ M^{-1}$).

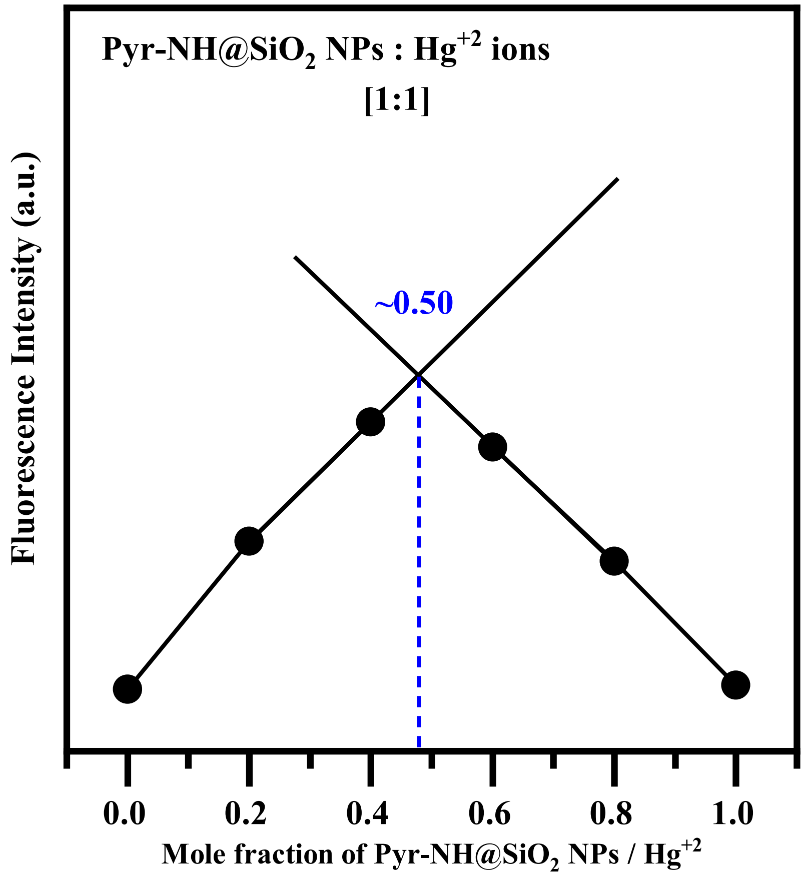

Figure S11: Job's plot for determining the binding stoichiometry of Pyr-NH@SiO_2 NPs and Hg^{+2} ions. The total concentration of Pyr-NH@SiO_2 NPs and Hg^{+2} ions was 5 µM. (λ_{ex} = 340 nm).
